# Mononuclear Aluminum–Fluoride Ions, AlF*_x_*^(+/−)^—Study of Plausible Frameworks of Complexes with Biomolecules and Their In Vitro Toxicity

**DOI:** 10.3390/molecules30020389

**Published:** 2025-01-17

**Authors:** Anja Pavlovič, Larisa Janžič, Lucija Sršen, Andreja Nataša Kopitar, Kathleen F. Edwards, Joel F. Liebman, Maja Ponikvar-Svet

**Affiliations:** 1 Department of Inorganic Chemistry and Technology, Jožef Stefan Institute, Jamova 39, SI-1000 Ljubljana, Slovenia; 2 Jožef Stefan International Postgraduate School, Jamova 39, SI-1000 Ljubljana, Slovenia; 3 Institute for Microbiology and Immunology, Faculty of Medicine, University of Ljubljana, Vrazov Trg 2, SI-1000 Ljubljana, Slovenia; larisa.janzic@mf.uni-lj.si (L.J.); lucija.srsen@mf.uni-lj.si (L.S.); andreja-natasa.kopitar@mf.uni-lj.si (A.N.K.); 4 Business Administration Program, School of Business, University of Maryland Global Campus, Largo, MD 20774, USA; kathleen.edwards@faculty.umgc.edu; 5 Department of Chemistry and Biochemistry, University of Maryland, Baltimore County, Baltimore, MD 21250, USA; jliebman@umbc.edu

**Keywords:** fluoride, aluminum, aluminum fluoride complexes, coordination, frameworks, toxicity, apoptosis, viability, THP-1 monocytes

## Abstract

The importance of fluorine and aluminum in all aspects of daily life has led to an enormous increase in human exposure to these elements in their various forms. It is therefore important to understand the routes of exposure and to investigate and understand the potential toxicity. Of particular concern are aluminum–fluoride complexes (AlF*_x_*), which are able to mimic the natural isostructural phosphate group and influence the activity of numerous essential phosphoryl transferases. Our review of salts of ionic AlF*_x_* species, which plausibly form the framework of complexes with biomolecules, revealed that the octahedral configuration of aluminum in the active site of the enzyme is preferred over the trigonal-bipyramidal structure. The effects of varying concentrations of fluoride, aluminum and AlF*_x_*—from micromolar to millimolar levels—on the viability and apoptosis rate of THP-1 monocytes were investigated using phosphate buffer solution as a culture media to simulate physiological conditions. Our results suggest that aluminum can reduce the direct toxicity of fluoride through the formation of AlF*_x_*. In view of the results found, further in vitro studies are required to clarify the toxicity mechanisms of these species.

## 1. Introduction

Fluorine is one of several trace elements that receives considerable attention due to its dual effect on human health. While fluorine (F) in its ionic form, fluoride anion (F^−^), has been recognized for nearly a century as beneficial for the prevention of dental caries at low concentrations, F^−^ at higher concentrations poses potential toxicity risks.

Various health authorities, including the European Food Safety Authority (EFSA) [1] and the Institute of Medicine (IOM) [2], consider the beneficial effects of F^−^ on the prevention of dental caries as crucial in establishing an adequate daily intake (ADI) of F^−^ from all sources, including non-dietary sources, of 0.05 mg per day per kilogram of body weight (bw) for all age groups over 6 months. In many high-income (developed) countries, F^−^ is intentionally added to public water supplies, salt and milk to increase its intake. In addition, various fluoride supplements are recommended to prevent dental caries. In these regions, mild dental fluorosis is often considered a cosmetic effect with little impact beyond the appearance [3]. Conversely, many citizens in low-income countries suffer from chronic fluoride poisoning [4,5,6,7] due to high natural F^−^ levels in drinking water [8], coal burning [9] and brick tea infusions [10]. This underscores the controversial nature of public water fluoridation in medicine, which is illustrated by the differing international views on water fluoridation. While the United States Centers for Disease Control and Prevention (US CDC) named water fluoridation as one of the ten great public health achievements of the 20th century in the US [11], in 2020, 72.7% of the US population connected to the municipal water supply system had access to fluoridated water [12]—many developed European [13] and Asian countries [14] opposed, stopped or banned water fluoridation.

**Figure 1:**
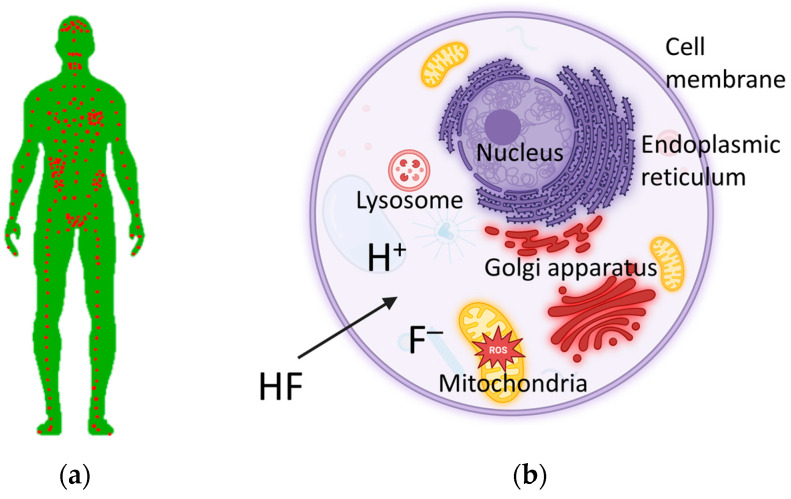
Different tissues and organs of a human body indicated by red dots (**a**), and the cellular organelles (**b**) most affected by toxic effect of F^−^.

Excessive intake of F^−^ over a long period of time can lead to skeletal fluorosis in children and adults. Both are ancient problems, as people have often settled in areas with high F^−^ concentrations in drinking water. A threshold of 0.03 mg per day/kg bw, which is below the ADI, has been proposed for the occurrence of dental fluorosis, but even this intake can lead to some degree of fluorosis in the population [3,15]. While fluorosis affecting calcified tissues is “only” a visible sign of excessive F^−^ intake over time, it is important to note that F^−^ can also damage almost all non-skeletal tissues (Figure 1a) [16]. Symptoms associated with chronic fluoride toxicity include (1) irritable bowel syndrome; (2) polyuria and polydipsia; (3) extreme fatigue/exhaustion/loss of muscle strength; (4) insomnia; (5) depression; (6) low hemoglobin; (7) high cholesterol and high blood pressure; (8) joint pain; (9) frequent bone fractures; (10) disabled children with bowed legs, knock-knee, short stature and mental retardation; and (11) pregnant women with anemia, low birth weight babies, premature births, intrauterine deaths, neonatal deaths and infant mortality [17,18,19].

Our current knowledge of the toxicity of F^−^ at the cellular level is based on in vivo and in vitro studies, including studies on oral, brain and blood cells, showing that F^−^ is capable of inducing apoptosis via both intrinsic and extrinsic pathways [20,21,22,23]. Fluoride can affect cell metabolism and cell physiology, depending on factors such as cell type, concentration and duration of exposure. At micromolar concentrations, F^−^ has been found to promote cell proliferation, while higher millimolar concentrations can inhibit enzymes, possibly triggering apoptotic cell death. Mechanisms underlying the toxicity of F^−^, which may also act in combination, include inhibition of metalloproteins, disruption of pH and electrolyte balance and disruption of cell organelles. Commonly observed phenotypes include oxidative stress, cell cycle arrest and apoptosis. Figure 1b shows a schematic representation of the cellular organelles most affected by F^−^ [22,23].

Less is known about the possible toxic effects of F^−^ in complexes with the metals aluminum (Al), beryllium (Be) or magnesium (Mg). Since the 1980s, these complexes (referred to for simplicity as AlF*_x_*, BeF*_x_*, MgF*_x_* to indicate the possible formation of a mixture of species) have been used as chemical probes for structural and mechanistic enzymatic studies—they are able to mimic the natural isostructural phosphate group and influence the activity of numerous essential phosphoryl transferases [24,25]. AlF*_x_* complexes are of particular concern because (1) the most physiologically active BeF*_x_* species is BeF_3_^−^, which has activity lower compared to AlF*_x_* species [26]; (2) unlike Al and Be, Mg does not spontaneously form stable (MgF_3_^−^) complexes with F^−^ in aqueous solutions [27].

AlF*_x_* complexes have been suggested to have the greatest potential for biological effects by mimicking gamma-phosphate, altering enzyme activity and activating guanine nucleotide-binding proteins (G-proteins), which are essential for endocrine and nervous system functions [28,29]. Their mechanism of interactions with biological molecules and enzyme binding sites have been well explored in theory [27,30] but in vivo and in vitro studies are scarce. A 2002 review article on the interactions between F^−^ and Al showed conflicting results regarding the effects of AlF*_x_* on tissue and organ structure, organ metabolism and enzyme activities [31]. Later studies reported similarly contradictory results. In vivo studies showed toxic effects of F^−^ and Al on neurons [32,33,34,35,36,37,38,39], testes and sperm quality [40] and hematologic parameters [41]. Conversely, no toxic effects of F^−^ and Al were observed on the femur, liver, brain [42] or kidney [42,43]. Al has also been suggested to reduce the toxicity of F^−^ [37]. An in vitro study in the rat pheochromocytoma cell line (PC12 cells) provided evidence of hormetic effects of NaF against Al maltotate; at low concentrations, F^−^ protects cells from Al toxicity and at high concentrations it enhances Al toxicity [44]. Enhanced apoptosis at high concentrations of F^−^ and Al was also observed in mouse neuroblastoma–rat glioma hybrid cells (NG108-15 cells) [45].

Epidemiological studies have investigated a possible link between F^−^ and Al concentrations in drinking water and the risk of dementia. A recent study suggests that elevated concentrations of F^−^ (*C* = 0.0534 ± 0.016 mg/L, range: 0.0238−0.0181 mg/L) and Al (*C* = 0.0374 ± 0.0100 mg/L, range: 0.0105−0.0928 mg/L) are associated with dementia risk in individuals consuming relatively low concentrations of both in drinking water [46]. In contrast, an early study conducted in three counties in the US reported that the county with the highest F^−^ concentration in drinking water (4.18 mg/L) had the lowest annual incidence of dementia, which was attributed to the fact that F^−^ attenuates the neurotoxicity of Al [47]. However, caution should be exercised when interpreting these results because, as with all epidemiological surveys, there is a possibility that the observed association is due to an unknown confounding variable.

This review of previous studies highlights a critical gap in our current understanding of the toxicity of AlF*_x_* and its mechanism and emphasizes the need for further investigation. Monocytes could serve as a promising model for in vitro immunotoxicity studies of F^−^ and AlF*_x_* and provide insights into potential immunomodulatory effects and inflammatory responses that are critical for understanding systemic effects. This is because monocytes, next to macrophages, play a central role in the recognition, phagocytosis and response to foreign substances by eliminating foreign and apoptotic cells and triggering inflammation through the production of reactive oxygen species (ROS) and the secretion of proinflammatory cytokines [48,49], which can lead to immunotoxicity when present in excess.

The aim of this study was to highlight the effects of human exposure to fluoride and its aluminum complexes by (1) providing a brief overview of the extent of human exposure to F^−^ and Al and their potential health effects, (2) reviewing the aqueous chemistry of F^−^ and Al and their complexes, (3) discussing well-defined salts of ionic AlF*_x_* species that plausibly form the framework of complexes with biomolecules, and (4) examining the effects of various concentrations of F^−^, AlF*_x_* and Al on apoptosis (programmed cell death) in THP-1 monocytes. It is anticipated that the results of this research will provide valuable insights for more focused research on potential immunomodulatory effects and inflammatory responses that are critical to understanding the systemic effects of AlF*_x_*.

## 2. Human Exposure to Fluorine and Aluminum

Fluorine and aluminum are highly abundant in the Earth’s crust. Both have found their use in human life. The beneficial effect of fluoride ion (F^−^) in the prevention of dental caries has been known for almost a century, and dental products are an important source of fluoride intake. Aluminum (referred to here as Al in any of its forms) is widely used in the packaging, construction, electronics, transportation and other industries due to its light weight, high strength and corrosion resistance. The general population is exposed to fluoride and aluminum through various sources such as water, food and beverages. Of concern are foods that are rich in both F^−^ and Al, such as tea, shellfish and cereals. A major source of both elements can be cookware. In addition, pharmaceuticals, consumer products, coal mixed with kaolinite and waste from cryolite alumina electrolysis are some other potential sources where F^−^ and Al can coexist.

### 2.1. Fluorine

Fluorine is highly electronegative and the most reactive element in the periodic table, which is why it is extremely rare in nature in elemental form. In the form of fluoride minerals such as fluorspar (CaF_2_), cryolite (Na_3_AlF_6_) and apatite (Ca_5_(PO_4_)_3_(OH,F,Cl)) as well as in groups of minerals, such as mica, hornblende and pegmatites such as topaz and tourmaline, it makes up 0.059% by weight of the Earth’s crust [50]. This makes it the 13th most common element and the predominant halogen.

The fluorine content in food is generally less than 50 μg/100 g on a fresh weight basis. Exceptions are (1) processed foods and beverages, which may contain significant amounts of fluorine if fluoridated water is used in their production; (2) tea infusions (typically between 0.31 and 8.9 mg/L) [51]; and (3) fish and shellfish (up to 150 μg/100 g fresh weight) [52]. Natural waters intended for human consumption contain trace amounts of up to a few mg/L, and some have even more toxic concentrations of F^−^. Artificially fluoridated water usually contains 0.5 to 1.0 mg/L of F^−^ [53]. Other systemic methods of fluoride delivery that consumers may consider are fluoridated salt (250–300 mg/kg fluoride) or milk (2.5–5 mg/L fluoride) and fluoride supplements (0.25–1.0 mg/unit fluoride). Dental products for topical use include toothpastes (250–2800 mg/kg), mouth rinses (230–900 mg/L), gels (1000–12,300 mg/kg) and varnishes (7000–22,600 mg/kg). Additional exposure may result from the metabolism of fluoride-containing compounds in pharmaceuticals, agrochemicals and materials.

Slightly soluble or insoluble salts of inorganic fluorides such as calcium fluoride are generally less toxic than more soluble salts such as sodium fluoride. The bioavailability of fluoride from various foods varies between 2 and 79% in adults [54]. About 20−25% of ingested F^−^ is absorbed from the stomach (pH ≤ 2) as hydrogen fluoride in a pH-dependent process, and about 75−80% of the remaining F^−^ is absorbed from the small intestine (pH ≈ 6.1–7.4) as F^−^ [55] in a pH-independent process [56,57,58]. In adults, about 50% of the daily fluoride intake is associated with the calcified tissues in the form of apatite, where about 99% of the F^−^ in the body is found. The remainder is distributed in the blood and soft tissues, where an equilibrium between extracellular and intracellular fluids is established within 24 h. The remaining 50% is mainly excreted via the kidneys. However, a damaged kidney impairs both the metabolism and the excretion of fluoride via the kidney, leading to further damage to the kidney [59].

In children, the estimated daily fluoride intake may be slightly higher than the ADI. In areas without fluoridation, the intake is between 0.019 and 0.062 mg/kg bw, while in fluoridated areas, it is between 0.015 and 0.064 mg/kg bw [16]. In regions with high natural F^−^ concentrations in water, beverages and food are the main sources of fluoride intake, which can lead to an intake up to six times higher than the ADI [16]. For adults living in non-fluoridated areas, the estimated daily fluoride intake is between 0.012 and 0.021 mg/kg bw for a 70 kg adult [16]. In fluoridated areas, this intake is almost twice as high and varies between 0.014 and 0.126 mg/kg bw [16].

### 2.2. Aluminum

Aluminum (Al) occurs mainly in the form of aluminosilicates, hydroxides, phosphates, sulfates and cryolite and makes up about 8.2% by weight of the Earth’s crust [50]. This makes it the third most abundant element after oxygen (46.1%) and silicon (28.2%) and the most abundant metallic element. Food is the main source of aluminum intake in humans, while the contribution of water is generally low. Unprocessed foods generally contain less than 5 mg/kg of aluminum. Higher concentrations, up to 10 mg/kg, are often found in bread, cakes and pastries, some vegetables, glacé fruits, dairy products, sausages, offal, shellfish, sugary foods, baking mixes and most flour-based products. Foods with very high average concentrations include tea leaves, herbs, cocoa and cocoa products and spices. An important source of aluminum exposure can be the use of aluminum-based cookware, especially when cooking acidic foods or when using scratched aluminum-based cookware with a non-stick coating. In addition, Al may also be present in certain pharmaceuticals and consumer products.

The bioavailability of Al is low (0.1–0.3% [60,61]). The presence or absence of dietary ligands in the intestine can influence the absorption of aluminum. Certain multidentate ligands such as citrate, lactate and other organic carboxylic acid complexing agents as well as fluoride can increase absorption, while others such as phosphate, silicate and polyphenols can decrease absorption [61]. After absorption, aluminum is distributed in all tissues, especially in the bones. It can penetrate the brain and reach the placenta and the fetus. Aluminum can remain in various organs and tissues for a very long time before it is excreted mainly (about 95%) via the kidneys, presumably as Al citrate [62]. Urinary excretion is of concern in conditions such as chronic kidney disease, where impaired kidney function can lead to reduced excretion of aluminum [63]. Chronic, routine exposure to aluminum is associated with diseases such as dialysis dementia, iron-related microcytic anemia, osteomalacia and possibly Alzheimer’s disease [64,65].

In contrast to fluoride, aluminum ions do not play a physiological role in metabolic processes, but can be toxic in high quantities, which is why the EFSA has set the tolerable weekly intake (TWI) at 1 mg/kg bw [61]. In non-occupationally exposed adults, large variations in total dietary exposure to aluminum have been found, both between and within countries and between different surveys. The mean daily exposure ranged from 1.6 to 13 mg, which corresponds to about 0.16 to 1.3 mg/kg bw per week for a 70 kg adult [61]. Dietary intake of aluminum is generally higher in children and they therefore represent the group with the highest potential exposure to aluminum per kg bw.

### 2.3. Aqueous Chemistry of Fluoride and Aluminum

The speciation of aluminum in aqueous solutions depends strongly on the pH value. Al^3+^ occurs as the octahedral hexahydrate Al(H_2_O)_6_^3+^ at a pH < 5.5 and as the tetrahedral aluminate Al(OH)_4_^−^ at a pH > 6.2 [66]. At a pH value between 5.5 and 6.2, various species form in a very cooperative manner [66]. The insoluble Al(OH)_3_ precipitates at a pH of 7.7 and the presence of fluoride increases the pH at which maximum precipitation is achieved [67].

The discovery that traces of aluminum are necessary for fluoride to exert its biological effects [68] has led to numerous investigations into the nature of the active AlF*_x_* complex. Many researchers have postulated AlF_4_^−^ as this species, but AlF_4_^−^ does not occur under any conditions in aqueous solution. In the absence of competing ligands, ^27^Al NMR experiments revealed that the octahedral nature of the coordination sphere is predominant for fluoroaluminate complexes such as AlF_2_^+^·4H_2_O, AlF_3_·3H_2_O, AlF_4_^−^·2H_2_O and AlF_5_^2−^·H_2_O in aqueous solution, for pH < 8 and for all [F]/[Al] ratios [69].

**Figure 2:**
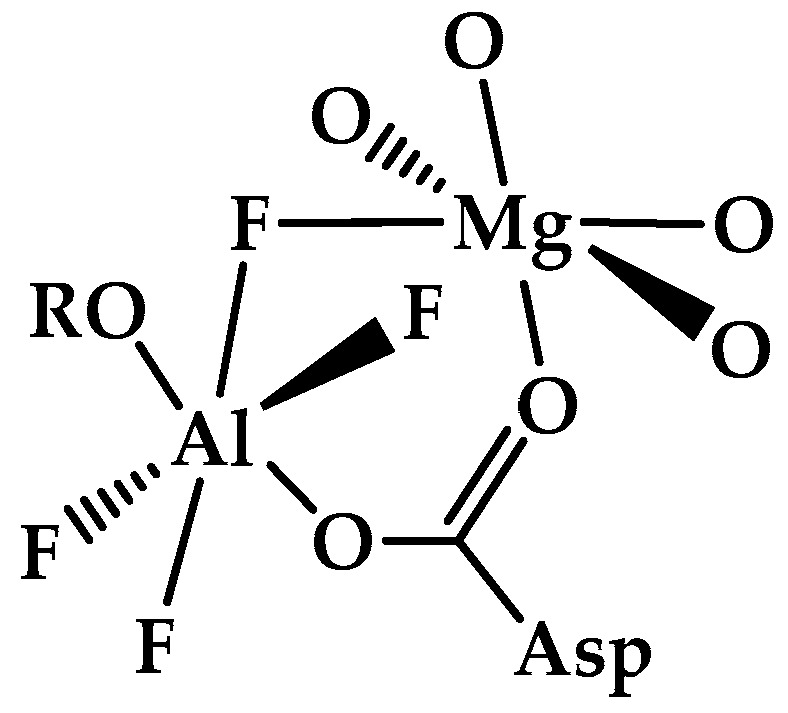
Typical aspartyl tetrafluoroaluminate substructure with catalytic magnesium coordination (adapted by the authors from [30]).

A number of crystal structures of nucleotide-binding proteins complexed with AlF_4_^−^ and AlF_3_ cores have been determined. It has been suggested that the transition between octahedral and trigonal bipyramidal structures is pH-dependent with a switch from AlF_4_^−^ to AlF_3_ at higher pH values [70]. However, the reliability of the AlF_3_ assignment has been questioned. An alternative interpretation, supported by solubility studies [67] and ^19^F NMR experiments, suggests that precipitation of aluminum at an elevated pH leads to replacement by magnesium in the protein complexes and a switch from octahedral to trigonal bipyramidal geometry [30]. Octahedral AlF*_x_* complexes include aspartyl tetrafluoroaluminates, nucleotide guanosine diphosphate (GDP) tetrafluoroaluminates, nucleotide adenosine diphosphate (ADP) tetrafluoroaluminates and complexes in which an aluminum trifluoride core is expanded to octahedral, six-coordination by having three oxygen ligands. The structures of these complexes, which are available in the Protein Data Bank (PDB), have been reviewed [30]. To illustrate the stereochemistry of octahedral AlF_4_^−^ complexes, a typical aspartyl tetrafluoroaluminate substructure with a coordinated catalytically active magnesium ion is shown in Figure 2 [30].

This brings us to the next section where we discuss well-defined species containing only F^−^ and Al that can plausibly form the scaffold of complexes with biomolecules.

## 3. Well-Defined Mononuclear Aluminum–Fluoride Ions (AlF*_x_*) Plausibly Forming the Framework of Complexes with Biomolecules

We begin with a brief discussion of the structure of aluminum- and fluorine-containing salts or more properly salts containing [AlF]*_x_*^3−*x*^ (*x* = 1–6) ions (referred to as AlF*_x_* for simplicity). In all cases, we consider in this paper only X-ray crystallographically determined structures as opposed to those found in solutions or melts that are also interesting and relevant. However, our aim is to discuss well-defined species composed of fluorine and aluminum only (without additional ligands such as OH^−^) that plausibly form the framework of complexes with biomolecules. Sadly, we omit a chemical discussion of the salts, and even better of the ions they contain. This is because thermochemical data on enthalpies, entropies and free energies of formation, solution and complexation are almost always lacking. Even the simpler conceptual quantity of equilibrium constants is also almost always unreported in the literature. We also acknowledge that while we always provide the identity of the counterion in our text, we do not list the composition of all species containing a given (AlF*_x_*) ion. We will not even ask what the precise structure of any of these ions is that would be determined by an X-ray crystallographic measurement of some appropriate salt, nor give any other structural information about any of these salts, many of which are minerals. Indeed, we will not attempt to generate a compositional phase diagram interrelating diverse (AlF*_x_*) species with their associated cation unlike such as found in ref. [71]. Finally, although we acknowledge the large electronegativity difference between Al and both O and F, we will not consider the bonding or electronic structure of the species we are discussing and will not attempt to distinguish between the highly ionic Al–F bonds and the more covalent, often dative Al–O bonds in any of the species of interest, whether they are the binary ions themselves, their hydrates or complexes with a biomolecule of interest.

The toxicity of fluoroaluminum (AlF*_x_*) species is generally understood in terms of the species of biochemically related organophosphates. Because such species almost always contain one, two or three linked phosphate groups (e.g., adenosine monophosphate, adenosine diphosphate and adenosine triphosphate, commonly known as AMP, ADP and ATP), we will limit our attention to species containing only one, two or three so-attached fluoroaluminate groups in our discussion. We note the following plausible reactions of [AlF*_x_*]^3−*x*^ with some unspecified oxygen-centered nucleophile that will be generically written as [NucO]^−^ without any concern as to its identity or even its charge. Such nucleophiles can be the oxygen in [OH]^−^, a phosphate in a nucleotide, the –O in a serine in an enzyme, or some transition state or even the oxygen in water with its partial negative charge as the nearly ubiquitous solvent. Schematically writing these nucleophilic oxygens as the [NucO]^−^ that follows, we otherwise do not discuss the following reactions:

Displacement reactions of the type [AlF*_x_*]^3−*x*^ + [NucO]^−^ → [AlF*_x_*_–1_ONuc]^3−*x*^ + F^−^ (1)

Addition reactions of the type [AlF*_x_*]^3−*x*^ + [NucO]^−^ → [AlF*_x_*ONuc]^3−*x*−1^ (2)

Some idiosyncratic reaction, i.e., unique to the specified choice of [AlF*_x_*]^3−*x*^ and [NucO]^−^ (3)

The reason we ignore such reactions is simple: the desired data are generally not available, and where they are available, they are incomplete. (For a discussion of the special case of mononuclear ions proceeding from [AlF_6_]^3−^ to [Al(H_2_O)_6_]^3+^, see the following references [72,73,74].

As part of a recent quantum chemical study, the Protein Data Bank (PDB) was systematically searched and statistically analyzed for noncovalent Lewis base/Lewis acid oxygen (lone pair)/aluminum (σ-/π-hole) O → Al interactions [75]. Numerous examples were found for these now-called “Triel bonds (TrB)”. In particular, the authors specifically studied those species involving directional contact of either of the Lewis acid fluoroaluminum species AlF_3_ and [AlF_4_]^−^ with the active sites of phosphoryl transfer enzymes, most importantly involving any of the following Lewis bases, glutamic acid and glutamine protein residues (GLU and GLN) and the phosphates of adenosine and guanosine diphosphates (ADP and GDP). As part of this investigation, the contemporary quantum chemical approaches were successfully used such as molecular electrostatic potentials (MEPs), quantum theory of atoms in molecules (QTAIM), non-covalent interaction plots (NCIplot) and natural bonding orbitals (NBOs).

### 3.1. The Fluoroaluminum [AlF]^2+^ Cation

**Figure 3:**
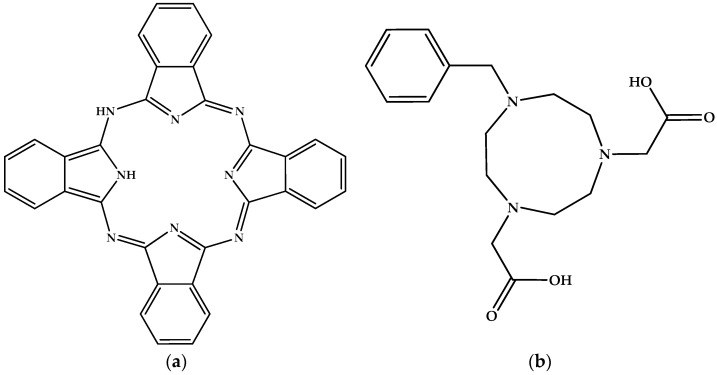
Structure of (**a**) phthalocyanine (CASRN = 574-93-6); (**b**) hexahydro-7-(phenylmethyl)-1*H*-1,4,7-triazonine-1,4(5*H*)-diacetic acid (CASRN = 934563-55-0).

The simplest aluminum fluoride ion is [AlF]^2+^. The fluorine free species [Al]^3+^ exists in numerous salts (e.g., diverse silicates that compose many common and less common minerals). Free, gaseous, unsolvated and thermodynamically stable [AlF]^2+^ ions have been observed [76]. We must admit that AlFO is not [AlF]^2+^[O]^2−^ and indeed, as a crystalline solid, it has both tetrahedrally and octahedrally coordinated aluminum [77], while as an isolated molecule, the description F–Al≡O has been suggested [78]. Relatedly, anhydrous binary salts such as AlFSO_4_, i.e., [AlF]^2+^[SO_4_]^2−^, remain unknown, in contrast to hydrates such as the pentahydrate, i.e., the mineral khademite [79]. A formal salt of [AlF]^2+^ is with phthalocyanine dianion [Pc]^2−^, AlFPc. Another formal derivative of [AlF]^2+^ is the chelate with 7-benzyl-1,4,-triazacyclononane-4,7-diacetic acid [80]. For additional information, we give the molecular structures of the related neutral species (Figure 3) instead of the Al derivatives, a practice we will continue with all other Al-containing species that follow.

Reaction chemistry of [AlF]^2+^ involves single-electron oxidants [81,82], halogen exchange [83], and axial-attachment reactions with diverse ligands [84]. Perhaps most relevant to our study is the Al–F polymerization (e.g., [81,85,86,87]), since it belies the simple [AlF]^2+^ description. We conclude our discussion of [AlF]^2+^ that diatomic doubly charged cations that are found as components of salts are surprisingly rare, e.g., [VO]^2+^[SO_4_]^2−^ (β-paulferite), in which there are both significantly short intra-cation and long inter-cation V–O bonds [88]. Stable triply charged diatomic cations are even rarer. For example, the reaction products of AlFPc with a single electron oxidant are best described as species with the modified ligand [Pc]^−^ rather than Al(IV), as found in the as yet unknown [AlF]^3+^.

### 3.2. The Difluoroaluminum [AlF_2_]^+^ Cation

**Figure 4:**
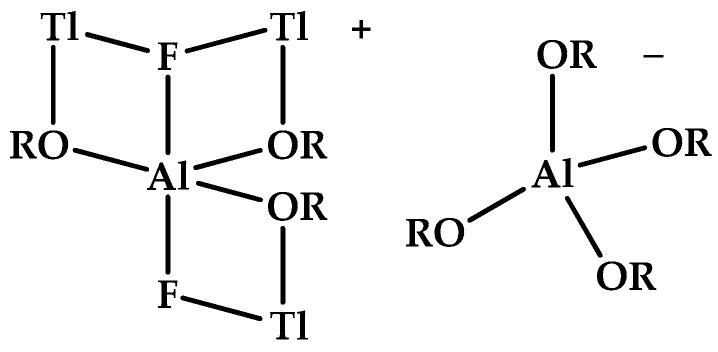
Schematic structure of aluminate(2−), tris(difluoromethanolato-κ*O*)difluoro-, trithallium(1+) (adapted by the authors from [95]).

The situation is similar with [AlF_2_]^+^ and its plausible anhydrous salts. To date, we are unaware of any crystalline species containing this triatomic fluoroaluminum cation [AlF_2_]^+^. However, there is evidence for solution phase species with the stoichiometries [AlF_2_]^+^[HSO_4_]^−^ and [AlF_2_]^+^[H_2_PO_4_]^2−^ [89]. This is further supported by the observation of the highly hydrated Al_2_SO_4_F_4_·12H_2_O [90]. In contrast to the doubly charged diatomic cations mentioned above, the [NO_2_]^+^ (nitronium) salts containing triatomic cations are well known and well-understood, as are those of [ClF_2_]^+^ and [BrF_2_]^+^ [91,92]. On the other hand, we know of no F^−^ addition product of the [AlF]^2+^ derivatives of phthalocyanine and of benzyltriazacyclononane-diacetic acid to form [AlF_2_]^+^ derivatives formally analogous to those of [ClF_2_]^+^ and [BrF_2_]^+^, i.e., [ClF_4_]^−^ and [BrF_4_]^−^ salts, respectively [93,94]. One species that can be understood simultaneously as an [AlF_2_]^+^ derivative and [AlF_5_]^2−^ analog is, [Tl_3_F_2_Al(OR)_3_]^+^[Al(OR)_4_]^−^ [R = CH(CF_3_)_2_] (Figure 4) [95].

### 3.3. The Aluminum Fluoride AlF_3_

**Figure 5:**
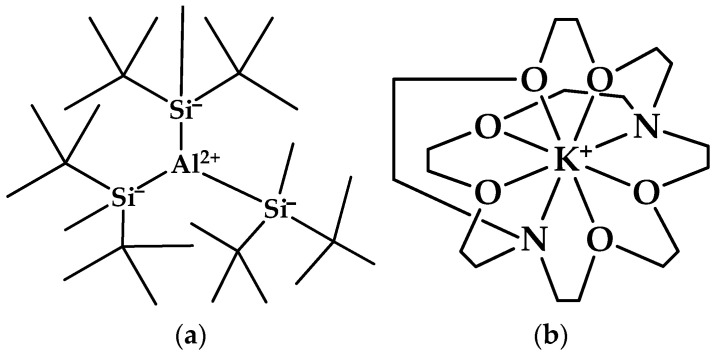
Structure of (**a**) tris[bis(1,1-dimethylethyl)methylsilyl]aluminate(1-) (CASRN = 853645-69-9); (**b**) (4,7,13,16,21,24-hexaoxa-1,10-diazabicyclo[8.8.8]hexacosane-κ*N*^1^,κ*N*^10^,κ*O*^4^,κ*O*^7^,κ*O*^13^,κ*O*^16^,κ*O*^21^,κ*O*^24^)potassium(1+) (CASRN = 61624-59-7).

We know enough to consider AlF_3_ not only as a neutral species. The corresponding radical ions cannot be isolated as salts and are therefore almost completely ignored in the current study. How could it be otherwise? We merely note that [AlF_3_]^+^ was investigated in the gas phase [96] and [AlR_3_]^−^ as a stable ion with R = di-*t*-butylmethylsilyl (Figure 5a) and K^+^[2.2.2]cryptand (Figure 5b) as the counterion [97].

We note now the existence of AlF_3_ as its trihydrate, or should we say trihydrates since there are two crystalline polymorphs. These would plausibly be the two isomeric fac- and mer- octahedral AlF_3_(H_2_O)_3_ discrete, hydrogen-bonded molecular species. In fact, the so-called α- and β-species are more properly described as randomly arranged Al(O,F)_6_ octahedra and ∞[AlF_2_F_2/2_(H_2_O)_2_]⋅H_2_O, respectively [98].

### 3.4. The Tetrafluoroaluminate [AlF_4_]^−^ Anion

Knowledge of the structure of isoelectronic anionic species such as [PO_4_]^3−^, [SO_4_]^2−^, and [ClO_4_]^−^, and related neutrals such as SiF_4_, suggests that this anion of interest, tetrafluoroaluminate(−1), should have a tetrahedral geometry. Many other approaches to molecular geometry such as hybridization and valence shell electron pair repulsion (VSEPR) make the same prediction. Indeed, this tetrahedral geometry is found in [(CH_3_)_4_N]^+^[AlF_4_]^−^ [99]. Interestingly, this binary salt readily and reversibly forms a monohydrate. The latter species may be described as idiosyncratic, as this monohydrate is the dimeric, or may we say more properly, binuclear salt 2[(CH_3_)_4_N]^+^{[AlF_4_(H_2_O)]}_2_^2−^. Both the anhydrous and the monohydrate salts have been crystallographically characterized. As mentioned above, the anhydrous tetrafluoroaluminate salt contains aluminum with the expected tetrahedral, four-coordinate, geometry. The binuclear salt contains edge-shared octahedral, six-coordinated aluminum. Octahedral, six-coordinated aluminum is the most common motif for Al^3+^ containing species. It is natural to ask what the coordination sphere around each aluminum in [AlF_4_(H_2_O)_2_]^2−^ is. We note that the statement that four fluorines and two oxygen atoms constitute the local environment of each aluminum does not define a unique structure. The two aluminum species can be bridged by two fluorine atoms, two oxygen atoms or one apiece. Experimentally, fluorine has been found to bridge the aluminums resulting in a crystal structure with extensive hydrogen bonding.

**Figure 6:**
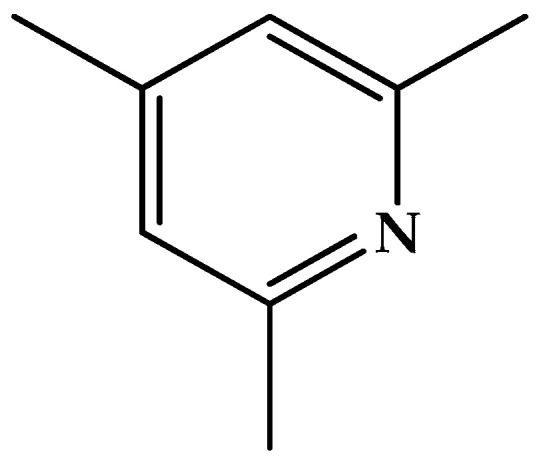
Structure of 2,4,6-trimethylpyridine (CASRN = 108-75-8).

Another salt of [AlF_4_]^−^ is with 2,4,6-trimethylpyridinium (Figure 6) as the chosen cation [100]. It is almost tempting to write “salts of [AlF_4_]^−^” because there are two independent structural types of this anion in this solid. The first is the tetrahedral pentatomic anion while the other is a polymer that contains extensively bridged aluminum–fluorine octahedra.

### 3.5. The Pentafluoroaluminate [AlF_5_]^2−^ Anion

**Figure 7:**
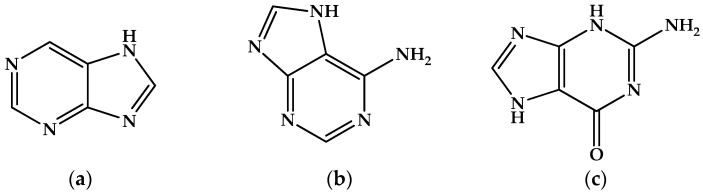
Structure of (**a**) purine (CASRN = 120-73-0); (**b**) adenine (CASRN = 73-24-5); (**c**) guanine (CASRN = 73-40-5).

The particularly toxicologically relevant salt with [AlF_5_]^2−^ is protonated purine, [Hpur]^+^_2_[AlF_5_]^2−^ [101]. We know that purine (Figure 7a) itself is the heterocyclic backbone of the DNA and RNA nucleobases, adenine (Figure 7b) and guanine (Figure 7c). Intriguingly, these three fluoroaluminate salts containing protonated purine, adenine and guanine have different stoichiometries and therefore different, interesting and irrelevant structures: [Hade]^+^_3_ [AlF_6_]^3−^·6.5H_2_O, [Hguan]^+^_3_[(Al_3_F_12_)]^3−^. However, the protonated purine salt [Hpur]^+^_2_[AlF_5_]^2−^ does not contain monomeric [AlF_5_]^2−^ ions, but rather a chain polymer with octahedral aluminum. So, what is the structure of the desired monomeric ion?

Hybridization and valence shell electron pair repulsion (VSEPR) theory-derived understanding plausibly suggest that [AlF_5_]^2−^ has a trigonal bipyramidal structure. This is sensibly found in the (CH_3_)_4_N^+^ salt [102], where the abstract of this paper begins with an exultant “Welcome to the family”. The same geometry can also be found in the tetraethylammonium salt [103]. This salt is the dihydrate and we acknowledge our surprise that it does not contain an octahedrally coordinated aluminum like [AlF_5_·H_2_O]^2−^. A crystallographically defined salt of this latter octahedral ion [AlF_5_·H_2_O]^2−^ is with K^+^ [104]. We may even ask why we ever even thought that [AlF_5_]^2−^ might not have a trigonal bipyramidal structure. We recall that this is the structure of its isoelectronic gas phase phosphorus analog PF_5_ [105,106,107]. However, the long-known, valence isoelectronic PCl_5_ has multiple crystalline forms [PCl_4_]^+^[PCl_6_]^−^ and (two variants of) 2[PCl_4_]^+^·[PCl_6_]^−^[Cl]^−^ [108,109,110]. In these examples, the cation and anion components of these species contain four- and six-coordinated phosphorus, respectively. It is only in the gas phase that we have discrete, i.e., monomolecular, trigonal bipyramidal PCl_5_ [107].

### 3.6. The Hexafluoroaluminate [AlF_6_]^3−^ Anion

The [AlF_6_]^3−^ anion is undoubtedly the most studied aluminum–fluorine-containing ion. This is not surprising—the solid sodium salt Na_3_AlF_6_ is commonly referred to as the mineral “cryolite” and is an essential part of the electrochemical Hall–Héroult process for the industrial production of elemental Al metal. The corresponding M_3_AlF_6_ salts with other alkali metals and [NH_4_]^+^ are also generally referred to as cryolites. The [AlF_6_]^3−^ ion, wherever it is found, contains an octahedral anion with its six Al–F bonds. As it follows from the VSEPR and hybridization logic, this ion is unequivocally octahedral with its six Al–F bonds, although we acknowledge that there is some lattice-induced distortion from a regular polyhedron. In this paper, we will not attempt to explain these structural variations nor the small structural changes and the crystal-type transition temperature as the M changes. Rather, we provide the interested reader with some relevant references that we have for M = Li [111], M = Na [112], M = K [113], M = Rb, Cs [114], M = NH_4_ [115].

In contrast to solids, it has been suggested [73,116] that [AlF_6_]^3−^ does not exist in aqueous solution, as opposed to ions in which some of the aluminum F^−^ has been replaced by [OH]^−^ and/or H_2_O. In other words, these fluorine–oxo exchange reactions are examples of our earlier displacement reaction type (1). However, in this paper, we will ignore thermochemical discussions of general ions of the type [AlF*_x_*(OH)*_y_*(H_2_O)*_z_*]^3−*x*−*y*^ regardless of the values of *x*, *y* and *z*. Relatedly, we know of no thermochemical discussion in the literature of [AlF_7_]^4−^, any of its oxygen-containing analogs or, for that matter, any discussion of seven-coordinated Al at all in the experimental literature.

We close with a conjecture about the polymorphism of [AlF_6_]^3−^ salts. We recognize polymorphism in salts can arise from different structural arrangements of the component atoms in a polyatomic ion. It is facilitated when the two arrangements have nearly the same interaction energy between the ions, both the stabilizing attraction between the ions of opposite sign and the destabilizing repulsion between those of the same sign. If an ion is spherical, then rotating it has absolutely no effect—we know enough to assume that F^−^ is spherical as opposed to tetrahedral with its four lone pairs. The four hydrogen atoms in an [NH_4_]^+^ ion form a tetrahedral shape. This arrangement is spherical enough that it may be mimicked by K^+^, a monoatomic positive charge of nearly the same size. It is only the octupole moment that distinguishes the spherical K^+^ from [NH_4_]^+^. Since ion–octupole interactions go as the long range 1/R^3^ ion–ion interactions, [NH_4_]^+^ and K^+^ are thermochemical mimics for both salts and gas phase complexes [117]. In this context, the octahedral arrangement of the fluorine atoms in the [AlF_6_]^3−^ salts results in even smaller inter-ion interactions other than that of simple Coulombic repulsion. (The isoelectronic neutral SF_6_ exhibits weak intermolecular effects, [118]). We recognize this anion as triply negatively charged with six fluorines. Changing the local environment has little effect on the resulting hexadecapole charge assembly. These changes in the position and orientation of [AlF_6_]^3−^ should be facile—we recognize this by a different name, crystal polymorphism.

## 4. Results and Discussion

Numerous studies have investigated the toxicity of fluoride (F^−^) and aluminum (Al) at the cellular level [20,21,22,23,24,25,26,27,28,29,30,31,32,33,34,35,36,37,38,39,40,41,42,43,44,45,46,47,48,49,50,51,52,53,54,55,56,57,58,59,60,61,62] In contrast, the toxicity of AlF*_x_* and the mechanism of their interactions with biological molecules and enzyme binding sites have been extensively explored in theory [27,30]. These theoretical models provide valuable insights into potential toxic effects, but in vivo and in vitro studies are sparse and the results inconsistent [31,32,33,34,35,36,37,38,39,40,41,42,43,44,45]. To our knowledge, there are only a few in vivo [46,47] and in vitro [24,28] studies investigating the direct effects of AlF*_x_* complexes on the immune system.

The immune system is a specialized, distributed network that is closely linked to other biological systems and serves as the most effective defense against the invasion of pathogens. The innate immune system is the body’s first line of defense that is used by the host immediately or within hours after encountering an antigen and primarily comprises phagocytic cells such as monocytes, macrophages and neutrophils [119]. Besides phagocytosis, an important function of innate immunity is the rapid recruitment of other immune cells to the site of infection and inflammation through the production of inflammatory mediators, such as cytokines and chemokines [120]. Monocytes are therefore an ideal target for studying the toxicity of chemicals, as they are present in the bloodstream and have the ability to differentiate into macrophages and monocyte-derived dendritic cells, allowing a comprehensive assessment of toxic effects across various stages of the immune response. This direct involvement in immune responses makes them highly susceptible to environmental stressors, including various chemicals such as fluoride (F^−^) and aluminum (Al). When exposed to these toxins, monocytes can exhibit a range of responses, such as changes in viability, phagocytic activity, cytokine production and oxidative stress [121].

In this study, we investigated and compared the toxicity of F^−^, Al and their AlF*_x_* complexes using an in vitro monocyte model. THP-1 monocytes were treated for 48 h with standard solutions of F^−^, Al or AlF*_x_* at various concentrations ranging from physiological concentrations found in blood to those still soluble in phosphate-buffered saline (PBS) (1–1200 µM). The PBS was used to ensure an appropriate pH and osmolarity very close to physiological conditions and to avoid cell stress or lysis that can occur in ultrapure water. This stable environment helps to achieve more accurate and reproducible results that reflect the actual biological responses of the cells studied.

The toxicity of F^−^, Al and their AlF*_x_* complexes to THP-1 monocytes was investigated using the Annexin V/Propidium Iodide assay and flow cytometry to assess plasma membrane permeability and integrity. This approach allowed us to determine the percentage of apoptosis among cells and to distinguish between viable, apoptotic and necrotic cells. Apoptosis, a programmed cell death, is an important indicator of cell health and can reveal early signs of cell damage caused by toxic substances. Annexin V (AV) is a protein that has a high affinity for phosphatidylserine (PS), a phospholipid that migrates from the inner to the outer leaflet of the plasma membrane in the early stages of apoptosis [122]. At this early stage, the cell membrane remains intact and prevents the penetration of impermeable dyes such as the DNA-binding dye propidium iodide (PI), while AV binds to the exposed PS. At a later stage of the apoptotic process, the cell membrane loses its integrity, allowing PI to enter the cell. This combination indicates that the cell is not only undergoing apoptosis, but also that its membrane integrity is compromised, which is a hallmark of late apoptosis or necrosis. Using the AV/PI assay, we were able to accurately detect and differentiate the percentage of early apoptotic (AV+/PI−) cells and the percentage of late apoptotic (AV+/PI+) cells. Cells that were positive only for PI staining were identified as necrotic (AV−/PI+), while cells that were negative for both AV and PI were classified as viable (AV−/PI−). This method is highly sensitive and specific and enables the detection of apoptosis even at low chemical exposure, which is crucial for understanding the dose–response relationship and establishing safe exposure limits. In addition, flow cytometry enables the rapid analysis of large numbers of cells, ensuring robust and statistically significant results [123].

**Figure 8:**
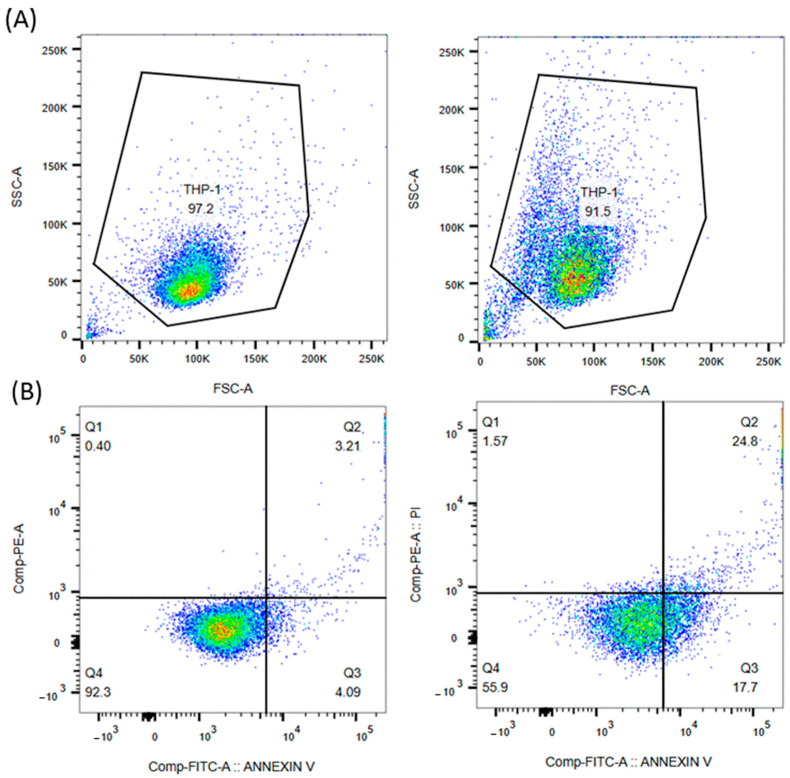
Flow cytometric analysis of THP-1 monocytes after 48 h of incubation with PBS (negative control, **left**) and 1200 µM F^−^ (**right**). (**A**) The scatter plots show the gating strategy for the negative control (**left**) and the cells treated with 1200 µM F^−^ (**right**). (**B**) The dot plots show the apoptosis profile for the negative control (**left**) and the 1200 µM F^−^-treated cells (**right**) (Q1: necrotic cells, Q2: late apoptotic cells, Q3: early apoptotic cells, Q4: viable cells). Blue/cool colors indicate low event density with few cells present, green to yellow represent moderate event density, orange to red indicate high-density regions with many concentrated cells and black/gray (optional) may highlight sparse or outlier events in less dense areas.

For a better understanding of the FITC Annexin V/Propidium Iodide method used in this study, the results of a representative experiment on the THP-1 monocyte cell line treated with the highest concentration of F^−^ (1200 µM) used in our experiments after 48 h of incubation are shown in Figure 8.

The results of the flow cytometric analysis show good agreement between the SSC-A vs. FSC-A plots (Figure 8A) and the apoptosis profiles (Figure 8B), indicating that the forward and side-scatter gating strategy employed is appropriate. As shown in Figure 8B, more than 90% of untreated cells (negative control) remain viable. However, when treated with 1200 µM F^−^, the percentage of viable cells (Q4) drops drastically to less than 60%, while almost 20% of cells undergo early apoptosis and 25% of cells undergo late apoptosis. The percentage of necrotic cells (Q1) is insignificant at less than 2%.

**Figure 9:**
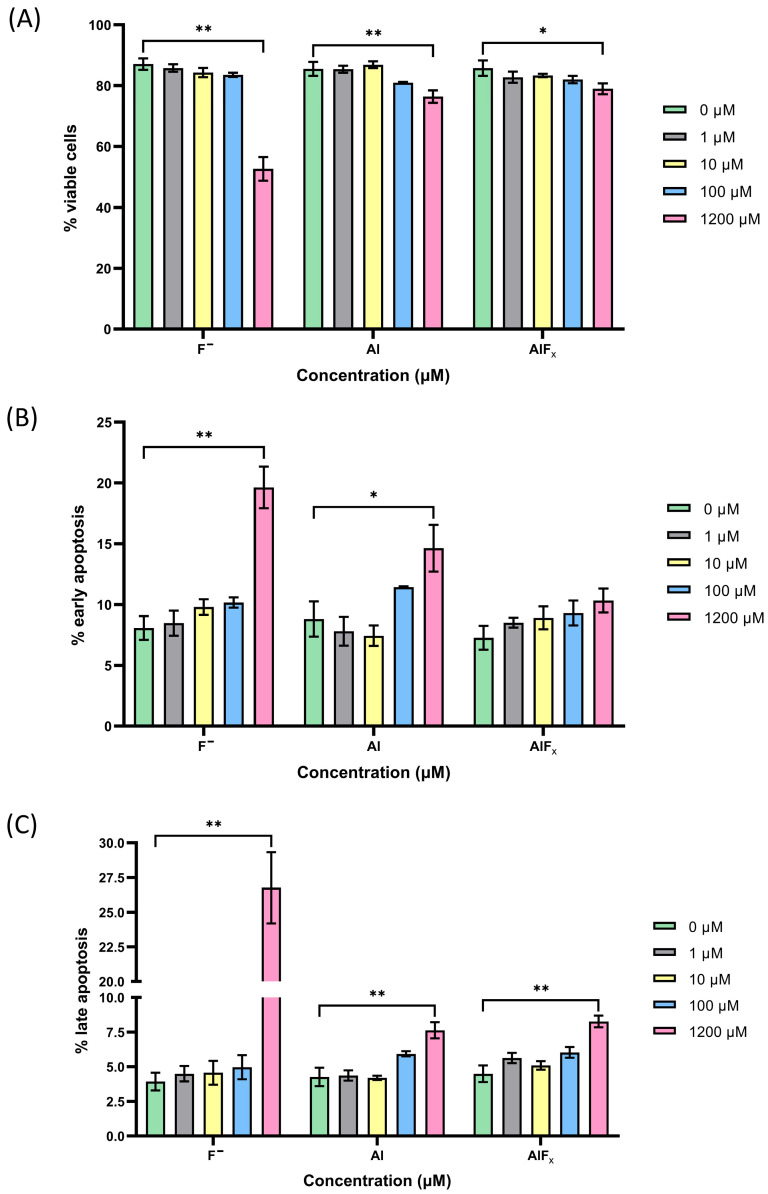
Viability of THP-1 monocytes exposed to four different concentrations of F^−^, Al or AlF*_x_* for 48 h by Annexin-V/Propidium Iodide assay. The bar graphs show the percentage of (**A**) viable (Q4) cells; (**B**) early apoptotic cells (Q3) and (**C**) late apoptotic cells (Q2). Data from three independent biological replicates are shown as means ± standard deviation (SD). ** *p* ≤ 0.01 and * *p* ≤ 0.05 compared to non-stimulated control cells, as determined by one-way ANOVA with post-hoc Šidák’s multiple comparisons.

Representative histograms of the flow cytometric analysis after exposure to different concentrations (1 µM, 10 µM, 100 µM and 1200 µM) of F^−^, Al or AlF*_x_* standard solutions for 48 h are shown in Figure 9. Plots (A), (B) and (C) represent the percentage of viable cells, early apoptotic cells and late apoptotic cells, respectively. Necrotic cells were excluded from the analysis as their percentage was negligible over the entire concentration range for all three standards analyzed.

The histograms visually show a cytotoxic response of THP-1 monocytes to increasing concentrations of the standard solutions of F^−^, Al and AlF*_x_* complexes, resulting in a loss of cell viability (Figure 9A) and an increase in the percentage of apoptotic cells (Figure 9B,C). A statistically significant decrease in viability was observed at the highest concentration (1200 µM) for all three standard solutions tested. Of the three standards, the F^−^ standard was found to be the most toxic (53% of viable cells), while Al and AlF*_x_* showed similar levels of toxicity with 76% and 79% of viable cells, respectively. Interestingly, at lower concentrations (1 and 10 µM), Al appeared to promote a slight but statistically insignificant increase in cell viability, an effect that was lost at 100 µM (Figure 9A). This slight increase may be due to the activation or stimulation of monocytes by aluminum.

Regarding apoptosis, a significant increase in both early and late apoptotic cells was observed at the 1200 µM concentration of F^−^ and Al; however, for AlF*_x_*, a significant increase was observed only in late apoptotic cells. F^−^ proved to be the most toxic among the standards tested, inducing early apoptosis in 20% of cells and late apoptosis in 27%. Al and AlF*_x_* showed similar levels of toxicity, inducing early apoptosis in up to 15% of cells and late apoptosis in 8% of cells (Figure 9B,C). Interestingly, Al again showed a unique pattern—a decrease in the percentage of early and late apoptotic cells was observed at lower concentrations of 1 and 10 µM and an increase at higher concentrations of 100 µM. This effect on cell viability was observed at concentrations of Al that are pharmacologically achievable in vivo [124], but further research is needed to understand the underlying mechanisms that drive these different responses to lower concentrations of Al.

Our results for F^−^ are consistent with previous studies reporting a significant decrease in cell viability and a significant increase in apoptosis following exposure of human RPMI8226 cells [125], primary cultured hippocampal neurons [126] and rat erythrocytes [127] to F^−^ at concentrations of 320 μM and higher. Previous studies on the toxicity of AlCl_3_ with AV/PI staining reported lower concentrations (10–100 µM AlCl_3_) for the onset of aluminum-induced cytotoxicity affecting the viability of peripheral blood mononuclear cells [128], Chinese hamster ovary (CHO) and CHO-XRS5 cells [129] and Chinese hamster V79 cells [130]. Remarkably, even the highest concentration of 300 µM AlCl_3_ [130] resulted in only a 20% reduction in cell viability compared to controls. The lower concentrations associated with Al toxicity and necrosis in these studies compared to our results may be attributed to the use of water media instead of the PBS culture media used in our research to simulate physiological conditions. The reason for this is that the phosphate contained in PBS media can form different complexes with aluminum, potentially leading to different toxicities and biological effects. In addition, aluminum can also form complexes with F^−^, which explains the comparable toxicity of Al and AlF*_x_*, i.e., the formation of AlF*_x_* complexes attenuates the toxicity of fluoride ions [37]. This proves that comparing the results of different studies can be challenging due to the different experimental conditions, cell types, measurement techniques and the complexity of translating in vivo results to in vitro contexts.

Fluoride and aluminum impair cell viability, disrupt the cellular redox balance and increase the formation of reactive oxygen species (ROS) and consequently increase oxidative stress in monocytes [131,132]. They have also been shown to alter the production of cytokines—particularly interleukins (IL-1β, IL-6), tumor necrosis factor-alpha (TNF-α) and interferon-gamma (IFN-γ)—suggesting shifts in inflammatory responses and possible links to chronic inflammation [133,134]. Therefore, future research is needed to analyze cytokine profiles, ROS levels, phenotype markers and gene expression to assess inflammation, metabolic changes and cell death after exposure to F^−^, Al and AlF*_x_*. This approach will allow us to compare their toxicity and uncover the underlying mechanisms of their toxic inflammatory effects.

## 5. Materials and Methods

### 5.1. Reagents and Materials

All reagents were of analytical quality. Ultrapure (Milli-Q) water (18.2 MΩ cm) from a Direct-Q 3 UV system (Merck Millipore, Darmstadt, Germany) was used for all experiments.

A phosphate-buffered saline (PBS) solution with a pH of 7.3–7.4 was prepared by dissolving 8.50 g sodium chloride (Fisher Scientific, Loughborough, UK), 2.21 g di-sodium hydrogen phosphate dihydrate (Honeywell, Seelze, Germany) and 0.34 g potassium dihydrogen phosphate (Carlo Erba, Milan, Italy) in a 1000 mL volumetric flask and diluted to the mark with water. Working standard solutions were prepared by appropriate dilution of 0.10 M standard solutions of F^−^ and Al prepared by dissolving NaF (Merck, Darmstadt, Germany) and AlCl_3_·6H_2_O (Chempur, Karlsruhe, Germany) in PBS buffer in 50 mL sterile polypropylene tubes.

### 5.2. Cell Line

The study was performed on an acute monocytic human leukemia cell line THP-1 cells (ATCC, Manassas, VA, USA). Cells were cultured in RPMI 1640 medium supplemented with 10 mM HEPES, 2 mM L-glutamine, 25 mM d-glucose, 1 mM sodium pyruvate (Gibco, Thermo Fisher Scientific, Waltham, MA, USA) and 10% fetal bovine serum (Sigma-Aldrich, St. Louis, MO, USA) in a humidified incubator under 5% CO_2_ and 37 °C. The cells were passaged every 3–4 days.

### 5.3. Apoptosis Assay

For fluorescence analysis, THP-1 monocytes were seeded in a 96-well plate at a density of 2 × 10^5^ cells/mL and treated in complete RPMI 1640 medium with 1 µM, 10 µM, 100 µM and 1200 µM final concentration solutions of F^−^, Al or AlF*_x_*. The cells were then incubated for 48 h.

After treatment, cells were collected, washed with PBS, resuspended in binding buffer and stained with Annexin V (AV) conjugated with fluorescein isothiocyanate (FITC) and propidium iodide (PI) using a BD Pharmingen^TM^ FITC Annexin V Apoptosis Detection Kit II (BD Biosciences, Franklin Lakes, NJ, USA). After 15 min incubation in the dark, the percentage of apoptosis was determined. The stained cells were immediately analyzed using the FACSCanto II flow cytometer (BD Biosciences, Franklin Lakes, NJ, USA). Data from 3 × 10^4^ cells were analyzed for each sample.

The specificity of AV binding to phosphatidylserine (PS) was assessed by pre-incubating the treated THP-1 cells with purified recombinant AV, which blocks the FITC AV binding sites.

AV and PI emissions were detected in the FL-1 (band pass 530 nm, band width 30 nm) and FL-2 (band pass 585 nm, band width 42 nm) channels, respectively. The cells were characterized by their forward/side scatter (FSC/SSC) parameters and included living cells with normal FSC/SSC parameters and dying cells with altered FSC/SSC. Cell debris characterized by a low FSC/SSC were excluded from analysis. Positioning of quadrants on AV/PI dot plots was performed as reported [135]. The results were expressed as a percentage of the 3 × 10^4^ cells.

### 5.4. Statistical Analysis

Data analysis was performed with BD FACSDiva v8.0.1 (BD Biosciences, Franklin Lakes, NJ, USA) and FlowJo v10.10 Software (BD Biosciences, Franklin Lakes, NJ, USA).

All data were tested for normal distribution using the Shapiro–Wilk or Kolmogorov–Smirnov test. The unpaired *t*-test or Mann–Whitney test was used to analyze the statistical significance of the data. ** *p* ≤ 0.01 and * *p* ≤ 0.05 were considered statistically significant. Statistical analysis was performed using GraphPad Prism Software v9.0 (GraphPad Software Inc., La Jolla, CA, USA).

Graphs were created using GraphPad Prism Software v10.0 or FlowJo Software v10.10, structures using CambridgeSoft ChemDraw Ultra 12.0 Software and images were created using Microsoft PowerPoint and BioRender Software (Science Suite Inc., Toronto, ON, Canada).

## 6. Conclusions

Our review of well-defined mononuclear aluminum fluoride (AlF*_x_*) ions that plausibly form the framework of complexes emphasizes that the octahedral coordination of aluminum (Al) is the most common structure for ions with the general formula AlF*_x_* and that the trigonal-bipyramidal structure is extremely rare. These findings align with previous studies [30,67], indicating that the octahedral configuration of Al is preferred in the active site of the enzyme. Therefore, the existence of a trigonal-bipyramidal configuration of Al in addition to the octahedral coordination within the active site of the enzyme remains questioned [70].

In this study, we observed a cytotoxic response of THP-1 monocytes to increasing concentrations (1–1200 µM) of standard solutions of fluoride (F^−^), Al and AlF*_x_* complexes. This response was characterized by a loss of cell viability and an increase in apoptotic cells, while the percentage of necrotic cells remained negligible. Our results suggest that Al may attenuate the toxicity of F^−^ by facilitating the formation of AlF*_x_* complexes. Interestingly, at lower concentrations (1 and 10 µM), the standard Al solution appeared to promote cell survival, in contrast to the effects of F^−^ and AlF*_x_*.

Given the increasing human exposure to F^−^ and Al from various sources and the limited experimental studies investigating the toxicity mechanisms of these species and the effects of AlF*_x_* species on cells and organisms, there is an urgent need for further research. Such studies are critical to understanding the potential health effects of aluminum and fluoride exposure, particularly with regard to public health and safety.

## Data Availability

The raw data supporting the conclusions of this article will be made available by the authors on request.
